# Efficacy of Ceftazidime-Avibactam Versus Polymyxin B and Risk Factors Affecting Clinical Outcomes in Patients With Carbapenem-Resistant *Klebsiella pneumoniae* Infections a Retrospective Study

**DOI:** 10.3389/fphar.2021.780940

**Published:** 2021-12-10

**Authors:** Jie Fang, Hui Li, Min Zhang, Guochao Shi, Mengying Liu, Yujie Wang, Xiaolan Bian

**Affiliations:** ^1^ Department of Pharmacy, Ruijin Hospital, School of Medicine, Shanghai Jiaotong University, Shanghai, China; ^2^ Department of Pharmacy Services, Boston Medical Center, Boston, MA, United States; ^3^ Department of Respiration and Critical Care Disease, Ruijin Hospital, School of Medicine, Shanghai Jiaotong University, Shanghai, China; ^4^ Institute of Respiratory Diseases, School of Medicine, Shanghai Jiaotong University, Shanghai, China; ^5^ Department of Pharmacy, Nanjing Drum Tower Hospital, The Affiliated Hospital of Nanjing University Medical School, Nanjing, China; ^6^ Department of Pharmacy, Shanghai 9th People’s Hospital, Shanghai Jiao Tong University School of Medicine, Shanghai, China

**Keywords:** carbapenem-resistant klebsiella pneumoniae, polymyxin B, Ceftazidime-avibactam, microbiological clearance, 28-day mortality

## Abstract

**Background:** The worldwide outbreak of carbapenem-resistant *Klebsiella pneumoniae* (CRKP) has become an urgent public health problem. High mortality and lack of effective treatments further pose new challenges to control this infection. However, studies about the evaluation of available antibiotics for CRKP infection are limited. The present study aimed to compare the efficacy of polymyxin B versus ceftazidime-avibactam (CAZ/AVI) in Chinese patients with CRKP infections and to identify risk factors affecting 7-day bacterial eradication and 28-day all-cause mortality.

**Methods:** From January 8, 2018, to July 6, 2020, a total of 115 adult CRKP infected patients from two tertiary teaching hospitals in Shanghai, China were enrolled based on the inclusion and exclusion criteria. By reviewing electronic medical records of these patients, demographic and clinical data were extracted. The selected patients were divided into polymyxin B and CAZ/AVI groups according to primary antibiotic exposure to compare therapeutic effects. Binary logistic and cox’s regression analysis were performed to identify risk factors for 7-day bacterial eradication and all-cause mortality.

**Results:** One hundred and five patients were treated with polymyxin B (67.8%) or CAZ/AVI (32.2%). Patients in the CAZ/AVI group had significantly lower rates of 28-day mortality (8.1 vs 29.5%, *p* = 0.013), higher microbiological eradication and 28-day clinical success. Multivariate analysis showed that Charlson comorbidity index (≥3) and prior antibiotic use within 90 days were independent risk factors for poor microbiological eradication. Cox’s regression analysis indicated that the length of hospitalization after CRKP infection and baseline creatinine clearance negatively affected 28-day mortality.

**Conclusion:** CAZ/AVI was more effective than polymyxin B and appeared to be a promising drug for CRKP infection, especially for critically ill patients.

## Introduction

In 1982, Carl Friedlander first described *Klebsiella pneumoniae* (*K. pneumoniae*), which belongs to the Enterobacteriaceae family and is ubiquitous in the environment, plant surface, or the animal mucosal surface. In humans, it is present within the gastrointestinal tract or the nasopharynx. It can cause many healthcare-associated infections, including pneumonia, urinary tract infections (UTIs), and bloodstream infections in immunocompromised and healthcare-exposed patients (Zhang et al., 2014; Martin and Bachman, 2018). Hypervirulent *K. pneumoniae* (hvKP), a distinct subtype of *K. pneumoniae*, commonly found in the Asian Pacific countries, can cause community-acquired and metastatic infections in immunocompetent and young healthy individuals (Thomas and Russo, 2019). These infections can lead to a higher incidence of liver abscesses, sepsis, pneumonia, necrotizing fasciitis, and meningitis. In China, the prevalence of hvKP is high, ranging from 8.33 to 73.9%, with a varied geographic distribution, suggesting an increasing level of concern and need for effective management (Zhang et al., 2016). Besides hvKP, the rate of carbapenem-resistant *Klebsiella* pneumonia (CRKP) has also dramatically increased worldwide in the past 20 years. The global dissemination of CRKP accounts for 60–90% carbapenem-resistant Enterobacteriaceae (CRE) infections in United States, Europe, and China. Significantly in China, the prevalence of CRKP has rapidly increased from 2.9% in 2005 to 25% in 2021 according to China Antimicrobial Surveillance Network (http://www.chinets.com/data/GermYear). A multicenter study, which covered 25 tertiary hospitals in 14 provinces of China, found that *K. pneumoniae* caused 73.9% of 664 CRE cases (Zhang et al., 2018a). Due to the limited therapeutic options, the mortality rate of CRKP-infected patients also increased to 40–50%. The predominant CRKP clone in China is ST11, and its high evolutionary rate led to the development of ST11-KL47 and ST11-KL64 strains with high virulence features and increased mortality rates (Zhou et al., 2020a).

Only a few antibiotics are active against CRKP. Historically, the best available therapies for CRKP include polymyxins, in combination with meropenem, imipenem, ceftazidime, or tigecycline. However, concerns for severe toxicity, such as nephrotoxicity and neurotoxicity, limit polymyxins use to a last-line option. In 2015 and 2016, the United States. Food and Drug Administration (FDA) approved ceftazidime/avibactam (CAZ/AVI) to treat complicated intra-abdominal infections (cIAI), complicated urinary tract infections (cUTI), hospital-acquired and ventilator-associated pneumonia (HAP/VAP). CAZ/AVI is an intravenously administered combination of the third-generation cephalosporine ceftazidime and the synthetic non-β-lactam β-lactamase inhibitor avibactam at a fixed ratio of 4:1. Ceftazidime exerts its antibacterial effect mainly by inhibiting peptidoglycan cross-linking in cell wall synthesis, inducing cell lysis and death. Avibactam has activity against Ambler class A, class C, and some class D β-lactamase, including Klebisella pneumonia carbapenemase (KPC), but does not inhibit metallo-β-lactamase (MBL) enzymes (Zhanel et al., 2013). In 2019, the National Medical Products Administration of China approved CAZ/AVI to treat cIAI, HAP, and VAP caused by multidrug-resistant Gram-negative bacteria.

The worldwide outbreak of CRKP, especially in China, high mortality rate, and lack of effective therapies have brought new clinical practice challenges. Several retrospective observational studies showed that CAZ/AVI-based regimens had greater mortality benefits against CRE isolates, predominately KPC-producing Klebisella pneumonia, than best available regimens including tigecycline, aminoglycosides, and polymyxins (Shields et al., 2017a; van Duin et al., 2018; Tumbarello et al., 2019; Tsolaki et al., 2020; Zheng et al., 2021). Most studies with polymyxins have compared the clinical outcomes of colistin-based therapy with CAZ/AVI. Colistin is administered as an inactive prodrug, colistin methanesulfonate (CMS). In contrast, polymyxin B is administered as an active drug. To our knowledge, the effective comparison between CAZ/AVI and polymyxin B for CRKP infection is scarce. In addition, data comparing the use of CAZ/AVI in the treatment of CRKP infection are limited in Chinese patient populations. This retrospective study aimed to evaluate the efficacy of CAZ/AVI with polymyxin B-based regimens in CRKP-infected patients and identify risk factors for 7-day microbiologic clearance and 28-day mortality.

## Methods

### Study Design, Setting, and Participants

We conducted a multicenter retrospective observational study at Ruijin Hospital (Shanghai, China), a 2100-bed tertiary teaching hospital, and Shanghai Ninth People Hospital (Shanghai, China), an 1800-bed tertiary teaching hospital, between January 8, 2018, and July 6, 2020. Inclusion criteria were as follows (Zhang et al., 2014): patients with a culture-confirmed CRKP infection and (Martin and Bachman, 2018) treated with polymyxin B or CAZ/AVI monotherapy or combination therapy. Exclusion criteria were: (Zhang et al., 2014): patients aged less than 16 years, and (Martin and Bachman, 2018) received antibiotic treatment for less than 24 h. This study involving human participants was reviewed and approved by the Research Ethics Commission of Ruijin Hospital (KY 2021–227). The Research Ethics Committee approved the study protocol and waived the informed consent because of the study’s retrospective nature. All demographic, clinical, and microbiological data were retrospectively extracted from the electronic medical records.

Eligible patients with CRKP infection were divided into the polymyxin B and CAZ/AVI groups based on primary antibiotic exposure. CAZ/AVI 2.5 g was administered intravenously every 8 h, and dose adjustments were made for patients with decreased renal function. Polymyxin B was administered at a dose of 1.25–1.5 mg/kg every 12 h after a loading dose of 2.0–2.5 mg/kg based on the International Consensus Guidelines for the Optimal Use of the Polymyxins. The primary outcomes include mortality on day 28 after the onset of the study infection, microbiological eradication, and clinical success rate. Risk factors for 7-day microbiological clearance and 28-day mortality of patients with CRKP infections were also analyzed.

### Definitions

Fever was defined as an axillary temperature of at least 38.3°C. Primary antibiotic exposure was defined as polymyxin B or CAZ/AVI exposure in patients with CRKP infection during the hospital stay. The severity of the disease was assessed by the Acute Physiologic Assessment and Chronic Health Evaluation (APACHE) II score on admission day (Knaus et al., 1985). Infection onset (day 1) was defined as the day that sample culture was drawn. Microbiological eradication/clearance was defined as the absence of the initially isolated pathogen from the site of index infection. Clinical success was defined as symptom resolution or significant improvement after the completion of antibiotic therapy on day 28 (Krapp et al., 2017). Hospital-acquired CRKP infection occurs 48 h or more after admission in non-intubated patients. The Charlson comorbidity index scores were used to predict the risk of mortality from disease (Charlson et al., 1994).

### Microbiology

*K. pneumoniae* were identified by matrix-assisted laser desorption ionization-time of flight mass spectrometer (bioMérieux, Marcyl’Étoile, France). Then imipenem, meropenem were used to screen for carbapenem resistant. Once non-susceptible to ≥1 carbapenem antibiotics, *K. pneumoniae* was identified as carbapenem resistant. Antimicrobial susceptibilities of meropenem, imipenem, ceftazidime, colistin, tigecycline and ceftazidime were tested by broth microdilution method using the VITEK 2 COMPACT (bioMérieux, Marcy-l’Étoile, France). Antimicrobial susceptibilities of ceftazidime-avibactam were acquired by the standard broth microdilution method following the criteria of the Clinical and Laboratory Standard Institute (CLSI) 2020 guideline. *Escherichia coli* ATCC 25922, *Pseudomonas aeruginosa* ATCC 27853 and *K. pneumoniae* ATCC 700603 were used as quality control strains in the antibiotics susceptibility assay. The MIC breakpoint for meropenem, imipenem, ceftazidime and ceftazidime-avibactam were interpreted based on CLSI 2020 criteria (CaLS institute, 2020). The minimum inhibitory concentration (MIC) breakpoint for tigecycline and colistin was interpreted in accordance with European Committee on Antimicrobial Susceptibility Testing (EUCAST) 2020 criteria (Satlin et al., 2020).

### Statistical Analysis

Statistical analyses were performed with SPSS software (version 25; SPSSInc., Chicago, IL, United States). Continuous variables with a normal distribution were expressed as mean ± SEMs and were analyzed by the Student’s t-test. Nonnormally distributed continuous variables were presented as median (interquartile range IQR) and analyzed by the Mann-Whitney U test. Categorical variables were expressed as n (%). The chi-square test or two-tailed Fisher’s exact test was used to compare the differences. A multivariate regression analysis model in a backward stepwise manner was performed to identify risk factors for microbiological clearance on day 7. Variables with a *p*-value ≤ 0.1 in univariate analysis were used in the binary logistic regression analysis. The Kaplan-Meier method was used for the survival analysis. All Variables with a *p*-value ≤ 0.1 were further used in Cox regression analysis model in a backward stepwise manner. Variables with a *p*-value ≤ 0.2 in polymyxin B and CAZ/AVI groups were included in the binary and Cox logistic regression analysis for adjustment of potential confounding factors. In contrast, variables with a *p*-value ≤ 0.1 were maintained in regression models. In addition, a propensity score for CAV/AVI group was calculated by the logistic regression models covering the variables with a *p*-value ≤ 0.1 as mentioned above. The propensity score was also included in the regression models. A *p*-value < 0.05 was considered statistically significant.

## Results

### Comparison of the Efficacy Between Polymyxin B and CAZ/AVI on CRKP Infected Patients

**FIGURE 1:**
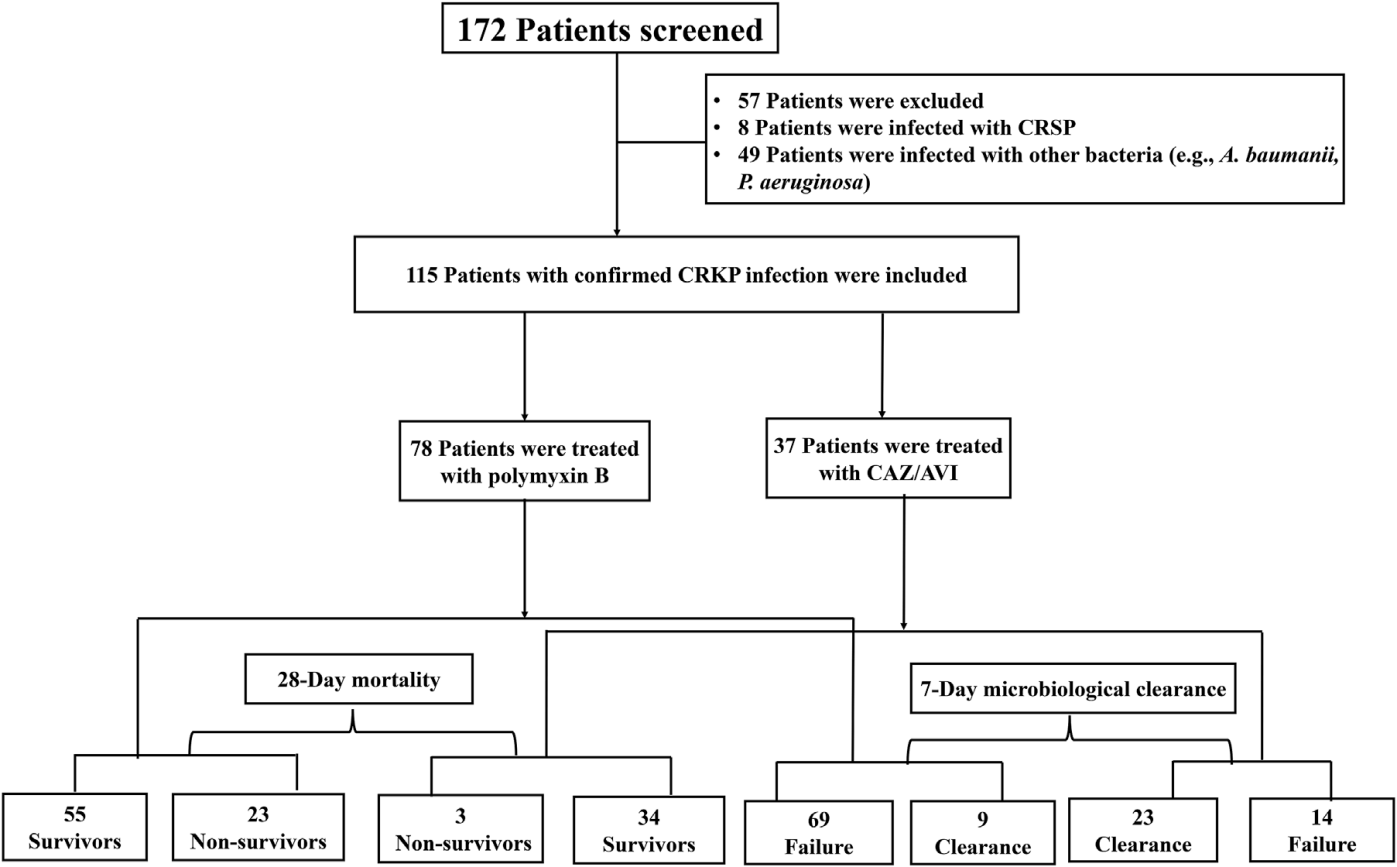
Flowchart of the patients included in the study. Abbreviations: CRSP, carbapenem-susceptible Klebsiella pneumonia; A. baumannii, Acinetobacter baumannii; P. aeruginosa, Pseudomonas aeruginosa; CRKP, carbapenem-resistant Klebsiella pneumonia; CAZ/AVI, Ceftazidime-avibactam.

During the study period, 115 eligible patients with CRKP infections who received either polymyxin B (*n* = 78, 67.8%) or CAZ/AVI (*n* = 37, 32.2%) were included in the analysis (Figure 1). Seventy-five and forty patients were from Ruijin Hospital and Shanghai Ninth People Hospital, respectively. The antibiotic susceptibility characteristics of *K. pneumoniae* were shown in Table1. Almost all *K. pneumoniae* isolates were resistant to meropenem, imipenem, ceftazidime but susceptible to colistin, tigecycline and CAZ/AVI.

**TABLE 1:** Antibiotic susceptibility characteristics of *K. pneumoniae* isolates.

Antimicrobial agent	MIC range (μg/ml)	S (%)	I (%)	R (%)
Colistin	0.125–0.5	100	N/A	0
CAZ/AVI	≤8/4−≥16/4	92.2	N/A	7.8
Tigecycline	≤0.5	100	N/A	0
Ceftazidime	4−≥64	0.88	6.19	93.81
Imipenem	≥16	1.77	2.65	95.58
Meropenem	≥16	0	0	100

Abbreviations: MIC, minimum inhibitory concentration; S, susceptible; I, intermediate; R, resistance; N/A, Not Applicable; CAZ/AVI, Ceftazidime-avibactam.

Table 2 summarizes the demographic and clinical characteristics of the study cohort. Patients ranged in age from 51 to 72 years. More than 90% of patients presented with hospital-acquired infection. Common infection types included pneumonia (57.4%) and bloodstream infections (50.4%). CAZ-AVI was most commonly used in combination with carbapenems (35.1%), aminoglycosides (13.5%), or tigecycline (20.9%). Polymyxin B group included carbapenem in 45.2%, aminoglycosides in 11.5%, and tigecycline in 5.1% of patient cases.

**TABLE 2:** Demographic and clinical characteristics of patients with CRKP infections.

	Total	Polymyxin B	CAZ/AVI	*p*-value
	(*n* = 115)	(*n* = 78)	(*n* = 37)	
Demographic characteristics				
Age	63 (51–72)	62.5 (52.5–70.5)	64 (47–72)	0.832
Gender				0.817
Female	27 (34.6%)	12 (32.4%)	39 (33.9%)	
Male	51 (65.4%)	25 (67.6%)	76 (66.1%)	
Weight (Kg)	65 (55–70)	65 (55–70)	65 (55–79.55)	0.672
BMI	23.11 (19.92–25.35)	22.72 (19.80–24.80)	23.7 (20.67–26.60)	0.424
Comorbidity				
Hypertension	51 (44.3%)	33 (42.3%)	18 (48.6%)	0.523
Malignancy	33 (28.7%)	26 (33.3%)	7 (18.9%)	0.11
Diabetes mellitus	31 (27%)	18 (23.1%)	13 (35.1%)	0.173
Cerebrovascular diseases	19 (16.5%)	10 (12.8%)	9 (24.3%)	0.121
Chronic kidney disease	19 (16.5%)	13 (16.7%)	6 (16.2%)	0.952
Chronic pulmonary disease	18 (15.7%)	13 (16.7%)	5 (13.5%)	0.664
Coronary disease	18 (15.7%)	11 (14.1%)	5 (18.9%)	0.507
Peptic ulcer	15 (13%)	12 (15.4%)	3 (8.1%)	0.279
Immunocompromised	12 (10.4%)	6 (7.7%)	6 (16.2%)	0.284
Chronic liver disease	10 (8.7%)	5 (6.4%)	5 (13.5%)	0.288
Solid organ transplantation	6 (5.2%)	1 (1.3%)	5 (13.5%)	0.021
Autoimmune disease	5 (4.3%)	4 (5.1%)	1 (2.7%)	0.915
Creatinine clearance (ml/min)	80.02 (44.37–124.08)	88.88 (49.12–140.91)	74.55 (39.26–112.38)	0.084
Severity variables				
Charlson comorbidity index	1 (0–3)	1 (0–2)	2 (1–4)	0.004
APACHE II score	15 ± 5.34	14.1 ± 5.51	13.68 ± 5.56	0.231
ICU administration	85 (73.9%)	56 (71.8%)	29 (78.4%)	0.453
Length of hospital stay before CRKP infection (days)	20 (7–35)	20 (8.75–34.25)	21 (2–40)	0.602
Length of hospital stay after CRKP infection (days)	31 (17–61)	30 (16.75–57.25)	40 (15–70.5)	0.76
Hospital stay (days)	60 (40–97)	56.5 (41.5–101.75)	64 (34.5–92)	0.94
Infection variables				
Hospital-acquired infection	109 (94.8%)	75 (96.2%)	34 (91.9%)	0.609
Number of infection site	2 (1–2)	2 (1–2)	1 (1–2.5)	0.75
Pneumonia	66 (57.4%)	43 (55.1%)	23 (62.2%)	0.476
Bloodstream	58 (50.4%)	44 (56.4%)	14 (37.8%)	0.063
Abdominal	32 (27.8%)	18 (23.1%)	14 (37.8%)	0.099
Urinary tract	12 (10.4%)	4 (5.1%)	8 (21.6%)	0.017
Other sites	15 (13.0%)	12 (15.4%)	3 (8.1%)	0.432
Concurrent fungal infection	29 (25.2)	22 (28.2%)	7 (18.9%)	0.284
Fever	80 (69.6%)	55 (70.5%)	25 (67.6%)	0.72
White blood cell count, x109 per L	10.57 (7.04–15.74)	11.42 (7.33–15.90)	10.02 (6.45–14.29)	0.215
Neutrophils count, x109 per L	8.77 (5.97–13.38)	9.23 (6.11–13.87)	8.7 (5.06–10.87)	0.288
Procalcitonin (μg/L)	2.3 (0.77–7.19)	2.695 (0.84–7.87)	1.8862 (0.35–4.48)	0.264
C-reactive Protein (mg/L)	90 (25.42–168)	95.2103 (25.70–174.82)	86.72 (23–164.3)	0.724
Prior healthcare history within 90 days of admission				
Hospitalization	74 (64.3%)	51 (65.4%)	23 (62.2%)	0.736
Surgery	54 (47%)	34 (43.6%)	20 (54.1%)	0.294
Antibiotic use	65 (56.5%)	42 (53.8%)	23 (62.2%)	0.401
Treatment characteristic				
Carbapenems	52 (45.2%)	39 (50%)	13 (35.1%)	0.135
Tigecycline	24 (20.9%)	19 (22.9%)	5 (15.6%)	0.39
Fosfomycin	19 (16.5%)	13 (16.7%)	6 (16.2%)	0.952
Cephalosporins	23 (20%)	19 (24.4%)	4 (10.8%)	0.09
Aminoglycosides	14 (12.2%)	9 (11.5%)	5 (13.5%)	0.762
Quinolones	13 (11.4%)	10 (13%)	3 (8.1%)	0.651
SMZ	8 (7.1%)	4 (5.1%)	4 (11.4%)	0.417
1 active antibiotic	20 (17.4%)	9 (11.5%)	11 (29.7%)	0.016
2 active antibiotic	51 (44.3%)	36 (46.2%)	15 (40.5%)	0.571
≥3 active antibiotic	44 (36.5%)	33 (42.3%)	11 (29.7%)	0.195
Duration of treatment (days)	16 (10–25)	17.5 (11–26)	15 (9–18)	0.047
Healthcare interventions				
Mechanical ventilation	74 (64.3%)	49 (62.8%)	25 (67.6%)	0.62
Vasoactive drugs	83 (72.2%)	55 (70.5%)	28 (75.7%)	0.564
ECMO	1 (0.9%)	1 (1.3%)	0 (0%)	0.678
CRRT	17 (14.8%)	9 (11.5%)	8 (21.6%)	0.155

Abbreviations: BMI, Body mass index; CRKP, carbapenem-resistant Klebsiella pneumonia; CAZ/AVI, Ceftazidime-avibactam; SMZ, compound sulfamethoxazole; ECMO, extracorporeal membrane oxygenation; CRRT, continuous renal replacement therapy.

**FIGURE 2:**
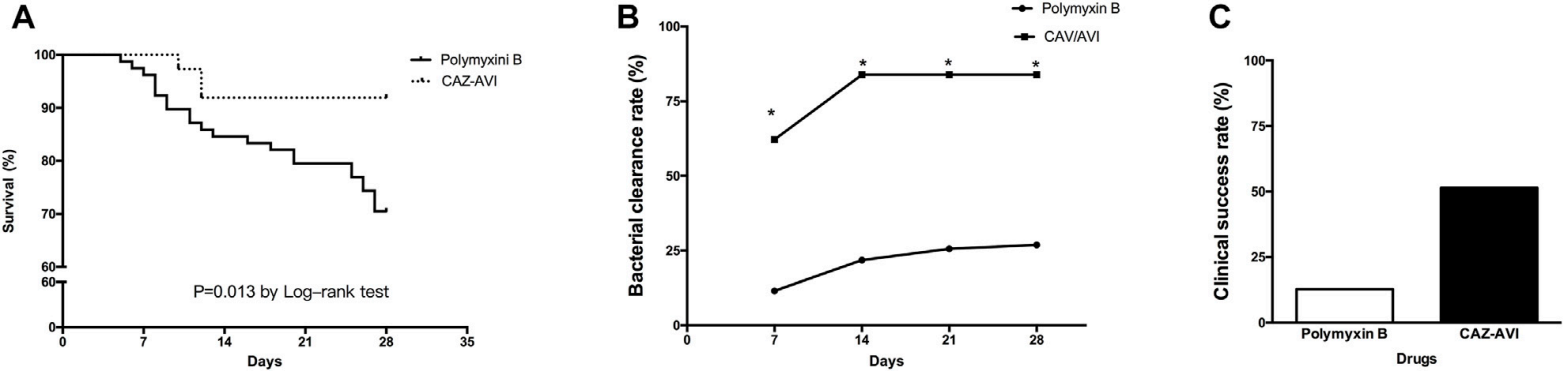
Efficacy of polymyxin B and CAZ/AVI on CRKP infected patients. Comparison of Kaplan-Meier survival curves **(A)**, 28-day bacterial clearance rate **(B)** and clinical success rate **(C)** between CRKP infected patients treated with polymyxin B and CAZ/AVI.

The 28-day mortality rates were 8.1 and 29.5% in the CAZ/AVI and polymyxin B groups, respectively. Survival analysis indicated that polymyxin B therapy was associated with a higher 28-day mortality rate than CAZ/AVI therapy (χ^2^ = 6.190, *p* = 0.013) (Figure 2A). 7-day microbiological clearance was accomplished in 62.2% of patients in the CAZ/AVI group and 11.5% of patients in the polymyxin B group (*p* < 0.001). 28-day microbiological clearance was accomplished in 83.8% of patients in the CAZ/AVI group, and 26.9% in the polymyxin B group (*p* < 0.001) (Figure 2B). The clinical success rate was observed in 51.4 and 11.5% in the CAZ/AVI and polymyxin B groups on day 28, respectively (Figure 2C).

### Risk Factors for 7-Day Bacterial Clearance in Patients With CRKP Infection

The microbiological clearance rates on day 7 were 62.2% for CAZ/AVI and 11.5% for polymyxin B groups, respectively. To identify the risk factors for bacterial eradication rate, we performed univariate and multivariable analyses in all enrolled patients. All CRKP infected patients were divided into failure and clearance groups according to the 7-day outcome of bacterial clearance. The demographic and clinical characteristics of these two groups were shown in Table 3. Statistically significant differences were found between Charlson comorbidity index (≥3), prior antibiotic use within 90 days and CAZ/AVI-based regimen. After adjusting the propensity score in binary logistic regression model evaluating risk factors for 7-day microbiological clearance, all variables remained in the model without significant difference (Table 4).

**TABLE 3:** Univariate analysis of factors associated with 7-Day microbiological clearance in the 115 patients with CRKP infection.

	Total	Failure	Clearance	*p*-value
	(*n* = 115)	(*n* = 83)	(*n* = 32)	
Demographic characteristics				
Age	63 (51–72)	64 (53–74)	61.5 (51–69)	0.851
Gender				0.948
Female	39 (33.9%)	21 (65.6%)	11 (34.4%)	
Male	76 (66.1%)	55 (66.3%)	28 (33.7%)	
Weight (Kg)	65 (55–70)	65 (55–70)	65 (55–71.13)	0.544
BMI	23.11 (19.92–25.35)	23.11 (19.76–25.39)	23.26 (20.42–25.25)	0.844
Comorbidity				
Hypertension	51 (44.3%)	25 (39.7%)	8 (53.3%)	0.336
Malignancy	33 (28.7%)	30 (36.1%)	3 (9.4%)	0.004
Diabetes mellitus	31 (27%)	22 (26.5%)	9 (28.1%)	0.861
Cerebrovascular diseases	19 (16.5%)	13 (15.7%)	6 (18.8%)	0.69
Chronic kidney disease	19 (16.5%)	12 (14.5%)	7 (21.9%)	0.337
Chronic pulmonary disease	18 (15.7%)	17 (20.5%)	1 (3.1%)	0.022
Coronary disease	18 (15.7%)	13 (15.7%)	5 (15.6%)	0.996
Peptic ulcer	15 (13%)	13 (15.7%)	2 (6.3%)	0.353
Immunocompromised	12 (10.4%)	9 (10.8%)	3 (9.4%)	1
Chronic liver disease	10 (8.7%)	6 (7.2%)	4 (12.5%)	0.461
Solid organ transplantation	6 (5.2%)	2 (2.4%)	4 (12.5%)	0.05
Autoimmune disease	5 (4.3%)	5 (6%)	0 (0%)	0.32
Creatinine clearance (ml/min)	80.01 (44.37–124.08)	89.78 (53.31–137.30)	67.83 (34.19–97.85)	0.007
Severity variables				
Charlson comorbidity index (≥3)	45 (39.1%)	41 (49.4%)	4 (12.5%)	<0.001
APACHE II score	14.1 ± 5.51	13.81 ± 5.72	14.88 ± 4.92	0.354
ICU administration	85 (73.9%)	63 (75.9%)	22 (68.8%)	0.434
Length of hospital stay before CRKP infection (days)	20 (7–35)	20 (8–34)	20 (4–41.5)	0.309
Length of hospital stay after CRKP infection (days)	31 (17–61)	27 (11–56)	42 (19.5–77.5)	0.722
Hospital stay (days)	60 (40–97)	56 (36–104)	68 (47–91.5)	0.897
Infection variables				
Hospital-acquired infection	79 (95.2%)	30 (93.8%)	109 (94.8%)	0.67
Number of infection site	2 (1–2)	2 (1–2)	1 (1–2.75)	0.601
Pneumonia	66 (57.4%)	47 (56.6%)	19 (59.4%)	0.789
Bloodstream	58 (50.4%)	47 (56.6%)	11 (34.4%)	0.032
Urinary tract	12 (10.4%)	8 (9.6%)	4 (12.5%)	0.736
Abdominal	32 (27.8%)	20 (24.1%)	12 (37.5%)	0.151
Other sties	15 (13%)	9 (10.8%)	6 (18.8%)	0.353
Concurrent fungal infection	29 (25.2%)	26 (31.3%)	3 (9.4%)	0.015
Fever	80 (69.6%)	57 (68.7%)	23 (71.9%)	0.738
White blood cell count, x109 per L	10.57 (7.04–15.74)	11.09 (6.74–16.19)	10.23 (7.29–14.54)	0.328
Neutrophils count, x109 per L	8.77 (5.97–13.38)	9.22 (5.97–14.07)	8.49 (5.96–10.94)	0.258
Procalcitonin (μg/L)	2.3 (0.77–7.19)	2.57 (0.85–7.43)	1.96 (0.44–6.55)	0.561
C-reactive Protein (mg/L)	90 (25.4173–168)	91 (26.85–161.00)	86.36 (20.18–183.75)	0.279
Prior healthcare history within 90 days of admission				
Hospitalization	74 (64.3%)	58 (69.9%)	16 (50%)	0.046
Surgery	54 (47%)	38 (45.8%)	16 (50%)	0.05
Antibiotic use	65 (56.5%)	52 (62.7%)	13 (40.6%)	0.033
Treatment characteristic				
CAZ/AVI	37 (32.2%)	14 (16.9%%)	23 (71.9%)	<0.001
Carbapenems	52 (45.2%)	40 (48.2%)	12 (37.5%)	0.302
Tigecycline	24 (20.9%)	19 (22.9%)	5 (15.6%)	0.39
Fosfomycin	19 (16.5%)	14 (16.9%)	5 (15.6%)	0.872
Cephalosporins	23 (20%)	19 (22.9%)	3 (12.5%)	0.212
Aminoglycosides	14 (12.2%)	10 (12%)	4 (12.5%)	1
Quinolones	13 (11.4%)	12 (14.6%)	1 (3.1%)	0.107
SMZ	8 (7.1%)	5 (6.1%)	3 (9.7%)	0.508
1 active antibiotic	20 (17.4%)	13 (15.7%)	7 (21.9%)	0.431
2 active antibiotic	51 (44.3%)	35 (42.2%)	16 (50.0%)	0.449
≥3 active antibiotic	44 (38.3%)	35 (42.3%)	9 (28.1%)	0.165
Duration of treatment (days)	16 (10–25)	17 (10–26)	15 (8.25–23.25)	0.256
Healthcare interventions				
Mechanical ventilation	74 (64.3%)	52 (62.7%)	22 (68.8%)	0.541
Vasoactive drugs	83 (72.2%)	60 (72.3%)	23 (71.9%)	0.965
ECMO	1 (0.9%)	1 (1.2%)	0 (0%)	1
CRRT	17 (14.8%)	9 (10.8%)	8 (25%)	1

Abbreviations: BMI, Body mass index; CRKP, carbapenem-resistant Klebsiella pneumonia; CAZ/AVI, Ceftazidime-avibactam; SMZ, compound sulfamethoxazole; ECMO, extracorporeal membrane oxygenation; CRRT, continuous renal replacement therapy.

**TABLE 4:** Binary logistic regression analysis of risk factors for patients with CRKP infection for 7-day microbiological clearance.

Variables	Without propensity score adjustment		Adjusted for the propensity score for CAZ-AVI-based treatment	
	*p*-value	OR (95% CI)	*p*-value	OR (95% CI)
charlson comorbidity index (≥3)	<0.001	0.061 (0.013–0.294)	0.002	0.074 (0.015–0.376)
Prior antibiotic use within 90 days	0.013	0.185 (0.048–0.704)	0.024	0.214 (0.056–0.820)
CAZ/AVI-based regimen	<0.001	44.094 (9.744–199.546)	<0.001	25.227 (5.278–120.573)

Abbreviations: CRKP, carbapenem-resistant Klebsiella pneumonia; OR, odds ration; CI, confidence interval, CAZ/AVI, Ceftazidime-avibactam.

All these statistically significant variables with *p* < 0.05 in univariate analysis were used for multivariate analysis. The results of binary logistic regression analysis indicated that Charlson comorbidity index (≥3) (OR = 0.074, *p* = 0.002) and prior antibiotic use within 90 days (OR = 0.214, *p* = 0.024) were independently associated with a lower rate of 28-day bacterial clearance. But patients treated with CAZ/AVI-containing regimens were associated with a higher 7-day microbiological clearance than polymyxin B, which was similar to our previous findings (OR = 25.227, *p* < 0.001). The results were shown in Table 3.

### Risk Factors for 28-Day All-Cause Mortality in CRKP Infected Patients

A total of 115 patients with CRKP infection were included in this analysis. These patients were classified into the survivor and non-survivor groups by the 28-day outcome. The overall all-cause 28-day mortality rate in CRKP-infected patients was 22.6% (26/115). The detailed analysis of demographic and clinical characteristics was summarized in Table 5. Kaplan-Meier survival analysis was used to distinguish statistically significant variables between survivor and non-survivor groups, which included age, chronic pulmonary disease, creatinine clearance, length of hospital stay after CRKP infection, hospital stay, 28-day microbiological clearance, CAZ/AVI-based regimen, and vasoactive drugs.

**TABLE 5:** Univariate analysis of factors associated with 28-Day mortality in the 115 patients with CRKP infection.

	Total	Survivors	Non-survivors	*p*-value
	(*n* = 115)	(*n* = 89)	(*n* = 26)	
Demographic characteristics				
Age	63 (51–72)	61 (49–69)	69 (63.75–76)	0.002
Gender				0.304
Female	39 (33.9%)	28 (31.5%)	11 (42.3%)	
Male	76 (66.1%)	61 (68.5%)	15 (57.7%)	
Weight (Kg)	65 (55–70)	65 (55–71.25)	63.5 (54.75–70)	0.618
BMI	23.11 (19.92–25.35)	23.11 (20.34–25.37)	22.55 (19.70–25.18)	0.642
Comorbidity				
Hypertension	51 (44.3%)	36 (40.4%)	15 (57.7%)	0.119
Malignancy	33 (28.7%)	24 (27%)	9 (34.6%)	0.448
Diabetes mellitus	31 (27%)	23 (25.8%)	8 (30.8%)	0.618
Cerebrovascular diseases	19 (16.5%)	17 (19.1%)	2 (7.7%)	0.281
Chronic kidney disease	19 (16.5%)	14 (15.7%)	5 (19.2%)	0.902
Chronic pulmonary disease	18 (15.7%)	10 (11.2%)	8 (30.8%)	0.035
Coronary disease	18 (15.7%)	14 (15.7%)	4 (15.4%)	0.966
Peptic ulcer	15 (13.0%)	12 (13.5%)	3 (11.5%)	0.545
Immunocompromised	12 (10.4%)	9 (10.1%)	3 (11.5%)	1
Chronic liver disease	10 (8.7%)	9 (10.1%)	1 (3.8%)	0.547
Solid organ transplantation	6 (5.2%)	6 (6.7%)	0 (0%)	0.391
Autoimmune disease	5 (4.3%)	3 (3.4%)	2 (7.7%)	0.686
Creatinine clearance rate (ml/min)	80.01 (44.37–124.08)	137.92 (90.99–200.49)	74.38 (54.03–111.88)	<0.001
Severity variables				
Charlson comorbidity index (≥3)	45 (39.1%)	34 (38.2%)	11 (42.3%)	0.706
APACHE II score	14 (10–18)	13 (10–18)	15 (10–18.25)	0.533
ICU administration	85 (73.9%)	63 (70.8%)	22 (84.6%)	0.158
Length of hospital stay before CRKP infection (days)	20 (7–35)	19 (6–34.5)	27 (16–49)	0.158
Length of hospital stay after CRKP infection (days)	31 (17–61)	46 (23.5–77.5)	10.5 (7.75–25)	<0.001
Hospital stay (days)	60 (40–97)	67 (47–110)	41 (25–59.5)	<0.001
Infection variables				
Hospital-acquired infection	109 (94.8%)	85(95.5%)	24 (92.3%)	0.886
Number of infection site	2 (1–2)	1 (1–2)	2 (1–2)	0.673
Pulmonary	66 (57.4%)	49 (55.1%)	17 (65.4%)	0.349
Bloodstream	58 (50.4%)	44 (49.4%)	14 (53.8%)	0.692
Urinary tract	12 (10.4%)	10 (11.2%)	2 (7.7%)	0.877
Abdominal	32 (27.8%)	26 (29.2%)	6 (23.1%)	0.539
Other sites	15 (13.0%)	12 (13.5%)	3 (11.5%)	1
Concurrent fungal infection	29 (25.2)	25 (28.1%)	4 (15.4%)	0.189
28-day microbiological clearance	52 (45.2%)	46 (51.7%)	6 (23.1%)	0.011
Fever	80 (70.2%)	63 (71.6%)	17 (65.4%)	0.543
White blood cell count, x109 per L	10.57 (7.04–15.74)	10.42 (6.88–14.68)	12.23 (8.24–16.49)	0.152
Neutrophils count, x109 per L	8.77 (5.97–13.38)	8.7 (6.01–11.99)	9.645 (5.7–14.87)	0.336
Procalcitonin PCT (μg/L)	2.3 (0.77–7.19)	1.89 (0.62–7.31)	2.94 (1.18–6.54)	0.44
C-reactive Protein (mg/L)	90 (25.42–168)	91 (29.93–177.24	85.33 (12.88–136.81)	0.22
Prior healthcare history within 90 days of admission				
Hospitalization	74 (64.3%)	58 (65.2%)	16 (61.5%)	0.734
Surgery	54 (47%)	43 (48.3%)	11 (42.3%)	0.589
Antibiotic use	65 (56.5%)	50 (56.2%)	15 (57.7%)	0.891
Antibiotic exposure during hospital stay				
CAZ/AVI-based regimen	37 (32.2%)	34 (38.2%)	3 (11.5%)	0.01
Carbapenems	52 (45.2%)	41 (46.1%)	11 (42.3%)	0.735
Tigecycline	5 (4.3%)	3 (3.4%)	2 (7.7%)	0.342
Fosfomycin	19 (16.5%)	14 (15.7%)	5 (19.2%)	0.902
Cephalosporins	23 (20%)	17 (19.1%)	6 (23.1%)	0.656
Aminoglycosides	14 (12.2%)	14 (15.7%)	0 (0%)	0.069
Quinolones	13 (11.4%)	12 (13.5%)	1 (4%)	0.336
SMZ	8 (7.1%)	7 (8%)	1 (3.8%)	0.767
1 active antibiotic	20 (17.4%)	17 (19.1%)	3 (11.5%)	0.368
2 active antibiotic	51 (44.3%)	38 (42.7%)	13 (50.0%)	0.422
≥3 active antibiotic	44 (38.3%)	34 (38.2%)	10 (38.5%)	0.921
Duration of treatment (days)	16 (10–25)	17 (11–26)	10.5 (8.75–22.5)	0.016
Healthcare interventions				
Mechanical ventilation	74 (64.3%)	54 (60.7%)	20 (76.9%)	0.128
Vasoactive drugs	83 (72.2%)	60 (67.4%)	23 (88.5%)	0.035
ECMO	1 (0.9%)	1 (1.1%)	0 (0%)	1
CRRT	17 (14.8%)	12 (13.5%)	5 (19.2%)	0.68

Abbreviations: BMI, Body mass index; CRKP, carbapenem-resistant Klebsiella pneumonia; CAZ/AVI, Ceftazidime-avibactam; SMZ, compound sulfamethoxazole; ECMO, extracorporeal membrane oxygenation; CRRT, continuous renal replacement therapy.

Variables with *p* < 0.1 were further analyzed using Cox regression analysis and presented in Table 5. The results suggested that the following factors independently decreased the 28-day mortality rate of CRKP infection: CAZ/AVI-based regimen, length of hospital stay after CRKP Infection, and creatinine clearance (OR = 0.989, *p* = 0.23). After adjusting the propensity score in Cox regression model evaluating risk factors for mortality, all variables remained in the model without significant difference (Table 6).

**TABLE 6:** Cox regression analysis of risk factors for patients with CRKP infection for 28-day mortality.

Variables	Without propensity score adjustment		Adjusted for the propensity score for CAZ-AVI-base treatment	
	*p*-value	OR (95% CI)	*p*-value	OR (95% CI)
CAZ/AVI-based regimen	0.005	0.178 (0.053–0.601)	0.003	0.153 (0.045–0.521)
Length of hospital stay after CRKP infection (days)	<0.001	0.928 (0.896–0.961)	<0.001	0.927 (0.893–0.926)
Creatinine clearance (ml/min)	0.019	0.99 (0.981–0.998)	0.023	0.989 (0.98–0.998)

Abbreviations: CRKP, carbapenem-resistant Klebsiella pneumonia; OR, odds ration; CI, confidence interval, CAZ/AVI, Ceftazidime-avibactam.

## Discussion

Infections attributed to CRKP are being increased and spread worldwide, posing significant threat to public health. According to two national surveillance reports from China, the prevalence of CRKP has been markedly increased since 2005 from 3.0 to 20.9% in 2017 (Hu et al., 2018). Risk factors for the development of CRKP infections included longer length of hospital stay, admission to ICU, previous antibiotic use, exposure to carbapenems (Liu et al., 2018). With the emergence and rapid expansion of carbapenem-resistant pathogens, including *K. pneumonia*, limited antibiotics (CAZ/AVI, polymyxins or tigecycline) are effective for treatment (van Duin et al., 2013). Clinical data comparing the effectiveness of ploymyxins versus CAZ/AVI in treating CRKP infection is limited. Van Duin and colleagues prospectively analyzed 137 CRE-infected patients to compare the clinical outcomes between colistin and CAZ/AVI-based regimens. The authors found that all-cause 30-days hospital mortality for CAZ/AVI and colistin-based regimens were 9 vs. 32%, respectively (van Duin et al., 2018). To our knowledge, the comparison between CAZ/AVI and polymyxin B-based regimens has not been reported in other retrospective studies^9-13^. The results of this retrospective study demonstrated that CAZ/AVI-containing regimen significantly decreased the 28-day mortality rate, increased 7-day microbiological clearance, and 28-day clinical success rate, compared with polymyxin B in patients with CRKP infection. Approximately 74% of patients included in the study were in the ICU. Our results revealed that CAZ/AVI was superior to the best available therapies, including carbapenem plus aminoglycoside, carbapenem plus colistin against CRKP infection, which were similar to other multicenter retrospective cohort studies^9-13^. Tsolaki and his colleagues conducted a retrospective observational study on 77 mechanically ventilated patients with CRE infections in ICU to evaluate clinical, microbiological, and safety outcomes. The authors showed that the CAZ/AVI-containing regime was more effective than other available antibiotics for treating CRE infections by increasing survival rate, microbiological eradication, and clinical cure rate (Tsolaki et al., 2020). In addition, a series of case reports also demonstrated that CAZ/AVI achieved treatment success in vertebral osteomyelitis, bloodstream, and pulmonary infections caused by CRKP in kidney transplant patients (Cani et al., 2018; Wang et al., 2020). Our results, retrospective cohort studies, and case reports indicated that CAZ/AVI was superior to polymyxin B for CRKP infected patients, especially for critically ill patients.

Studies are conducted *in vitro* and vivo to compare CAZ/AVI monotherapy with combination therapy. Zheng and his colleagues suggested that CAZ/AVI combined with another *in vitro* non-susceptible antimicrobials could significantly decrease the 30-day mortality rate of critically ill patients with CRKP infections compared with CAZ/AVI monotherapy (Zheng et al., 2021). An *in vitro* study indicated that polymyxin B and CAZ/AVI combinations could improve the antimicrobial activity, delay or suppress the regrowth of CRKP resistant subpopulation (Ma et al., 2019). However, results of meta-analysis indicated that there were no siginifaicant difference in mortality rate, microbiologically negative and clinical success between CAZ/AVI-based combination tehrapy and CAZ/AVI monotherapy (Li et al., 2021). Therefore, the actual effect of combination therapy on CRKP infection in humans is unknown. Future studies to compare the efficacy of monotherapy (polymyxin B or CAZ/AVI) with combination therapy in CRKP infected patients are warranted.

Although CAZ/AVI is a novel antibiotic to CRKP infection, 23.3% of CRKP strains isolated from neonates were resistant to CAZ/AVI (Zhou et al., 2020b). And the resistance rate of CAZ/AVI was about 3.7% (32/872) in China. Among the 32 resistant isolates, 53.1% were MBL-producing *K. pneumoniae*, 40.6% were carbapenemase-producing *K. pneumoniae*, the remaining were a mix of both (Zhang et al., 2020a). CAZ/AVI resistant *K. pneumoniae* usually emerged in patients after 10–19 days of CAZ/AVI treatment. By whole-genome sequencing, the mutations of plasmid-bone bla_kpc-3_ in CAZ/AVI resistant *K. pneumoniae* were not detected in control baseline isolates (Shields et al., 2017b). The increased use of tigecycline and colistin also led to the emergence of tigecycline- and colistin-resistant CRKP strains (Zhang et al., 2018b). A cohort study of 264 patients with CRKP infection found that colistin-resistant is up to 13% (Rojas et al., 2017). Nearly all CRKP strains isolated in our study are susceptible to colistin and CAZ/AVI. Once CRKP strains are resistant to these “last-line” treatments, the mortality would sharply increase. In order to control the worldwide outbreak of CRKP, prompt and appropriate antibiotic therapy for CRKP infection is necessary.

Several studies indicated the higher rates of treatment failure among people infected with CRKP isolated. In a multivariate analysis of risk factors for treatment failure of polymyxin B monotherapy, baseline renal insufficiency was associated with increased clinical failure in treating CRKP infection (Dubrovskaya et al., 2013). Risk factors for CAZ/AVI treatment failure and resistance among patients with CRE infection included pneumonia and renal replacement therapy (Shields et al., 2018). The present study showed that Charlson comorbidity index (≥3) and prior antibiotic use within 90 days were associated with decreased 7-day microbiological clearance. On the contrary, CAZ/AVI-based regimen increased the 7-day microbiological clearance rate and reduced the 28-day mortality rate. Moreover, the lower rate of 28-day all-cause mortality was positively related to the length of hospital stay after CRKP infection and baseline creatinine clearance. One retrospective analysis suggested that imipenem MICs of >8 mg/L, tigecycline therapy, and inappropriate treatment were associated with higher 28-day mortality in nontransplant patients infected with CRKP (Xiao et al., 2020). Therefore, appropriate antibiotics and an adequate treatment period would increase the rate of treatment success. A systematic review and meta-analysis of the mortality of patients infected with CRKP suggest that bloodstream infection, ICU administration, or solid organ transplantation were also strongly associated with higher mortality of CRKP infection (Xu et al., 2017).

As reported, carbapenem resistance is often associated with resistance to all traditional β-lactans and other antibiotics. The major mechanisms of carbapenem resistance are production of carbapenemases, production of efflux pumps, porin mutation or loss (Durante-Mangoni et al., 2019). The rapid increase of carbapenems resistance is associated with the most potent mechanism of carbapenemase such as New Delhi metallo-β-lactamase (NDM) especially NDM-1 which belong to Ambler class B1 superfamily. The percentage of NDM producer in MBL-producing CRKP is as high as 83.3% (Qamar et al., 2021). Molecular analysis of NDM-1gene in clinical isolates of Enterobacteriaceae form two tertiary hospital in Sakaka, Saudi Arabia found that *bla*_NDM-1_ and *bla*_NDM_ harbored isolates were respectively accounted for 63.2 and 36.8% (Ejaz et al., 2020). In asian padiatric patients, carbapenem resistance were accounted for 24% of total *E. coli* and the NDM producing *E. coli* strains harbored more NDM-5 (50%) than NDM-1 (46%) and NDM-4 (3.5%)variants (Nosheen et al., 2020; Nosheen et al., 2021). Until now, multiple NMD-1 inhibitors have been designed and tested, but there is still no inhibitor on market. In China, the primary resistant mechanism can be attributed to the production *K. pneumoniae* carbapenemase by ST11 clones (Wang et al., 2018; Tian et al., 2019; Wyres et al., 2020). These strains are hypervirulent, multidrug-resistant, and highly transmissible, posing a greater threat to public health (Gu et al., 2018) and dominantly harbored the carbapenemase gene bla_kpc-2_ (Yu et al., 2019; Lin et al., 2021). Among the KPC-2-producing ST11, carbapenem-resistant hypervirulent were more frequently identified than previously assumed, increasing from 2.1% in 2015 to 7.0% in 2017 and posed further infection control (Zhang et al., 2020b). But in the United States and Europe, the dominant sequence type of carbapenem-resistant strains of the *K. pneumoniae* are ST258, which are associated with high mortality by adopting two opposing infection programs through easily acquired gain- and loss-of-function mutations (Ernst et al., 2020). Therefore, genotypic identification of CRKP is beneficial to explore the resistant mechanism. We did not perform CRKP genotyping in this study. Thus, the prevalent strains responsible for CRKP infection in the two study sites were not identified. This was one limitation for our study. CAZ/AVI was not covered by most insurance plans and has not been widely used for critically ill patients in China. Only 32% of patients were in the CAZ/AVI group, which was another limitation for this study. Not all reported risk factors were identified given a relatively small number of non-survivor in the study. Further studies with a larger group of patients are warranted to identify additional risk factors associated with the 28-day mortality rate and 7-day microbiological clearance.

## Conclusions

In conclusion, our study showed that CAZ/AVI was more effective in treating CRKP infection than polymyxin B therapy, especially for critically ill patients. Charlson comorbidity index (≥3) and prior antibiotic use within 90 days were independent risk factors for inadequate bacterial eradication. Patients receiving CAZ/AVI-based regimens presented more rapid bacterial clearance and, most importantly, have increased survival compared to patients in the polymyxin B group. The length of hospitalization after CRKP infection and baseline creatinine clearance negatively affected 28-day mortality. Further large-scale clinical trials are needed to determine optimal CAZ/AVI therapies in treating CRE infections.

## Data Availability

The data supporting the conclusion of this article will be made available from the corresponding author upon on reasonable request.
